# Supplementary material for: Effects of various factors on Doppler flow ultrasonic radial and coccygeal artery systolic blood pressure measurements in privately-owned, conscious dogs
Source: PeerJ. 2017 Mar 22;5:e3101. doi: 10.7717/peerj.3101 (PMC5364917; doi:10.7717/peerj.3101)
Supplement: Table S1 [file peerj-05-3101-s001.docx]

| **Breed** | **Number of dogs** |
| --- | --- |
| Alaskan malamute | 1 |
| Basset hound | 1 |
| Beagle | 2 |
| Bernese mountain dog | 1 |
| Border collie | 2 |
| Bouvier des Flandres | 1 |
| Cardian Welsh corgi | 1 |
| Cavalier King Charles Spaniel | 1 |
| Chihuahua | 1 |
| Cocker spaniel | 1 |
| Collie | 2 |
| Dachshund | 2 |
| Dalmation | 1 |
| Golden retriever | 1 |
| Great pyrenees | 3 |
| Greyhound | 1 |
| Italian greyhound | 1 |
| Jack Russell terrier | 1 |
| Labrador retriever | 3 |
| Maltese | 1 |
| Mixed breed | 24 |
| Pomeranian | 2 |
| Pug | 1 |
| Rat terrier | 1 |
| Red bone hound | 1 |
| Shiba inu | 1 |
| Shih tzu | 1 |
| Standard poodle | 1 |
| Unassigned | 1 |
| West Highland white terrier | 1 |
| **Total** | **62** |
